# Obesity and COVID-19: Molecular Mechanisms Linking Both Pandemics

**DOI:** 10.3390/ijms21165793

**Published:** 2020-08-12

**Authors:** Andreas Ritter, Nina-Naomi Kreis, Frank Louwen, Juping Yuan

**Affiliations:** Division of Obstetrics and Prenatal Medicine, Department of Gynecology and Obstetrics, University Hospital Frankfurt, J.W. Goethe-University, Theodor-Stern-Kai 7, D-60590 Frankfurt, Germany; Nina-Naomi.Kreis@kgu.de (N.-N.K.); Frank.Louwen@kgu.de (F.L.)

**Keywords:** COVID-19, obesity, adipose-derived mesenchymal stem/stromal cells, cytokine storm, immune response, inflammation

## Abstract

The coronavirus disease 2019 COVID-19 pandemic is rapidly spreading worldwide and is becoming a major public health crisis. Increasing evidence demonstrates a strong correlation between obesity and the COVID-19 disease. We have summarized recent studies and addressed the impact of obesity on COVID-19 in terms of hospitalization, severity, mortality, and patient outcome. We discuss the potential molecular mechanisms whereby obesity contributes to the pathogenesis of COVID-19. In addition to obesity-related deregulated immune response, chronic inflammation, endothelium imbalance, metabolic dysfunction, and its associated comorbidities, dysfunctional mesenchymal stem cells/adipose-derived mesenchymal stem cells may also play crucial roles in fueling systemic inflammation contributing to the cytokine storm and promoting pulmonary fibrosis causing lung functional failure, characteristic of severe COVID-19. Moreover, obesity may also compromise motile cilia on airway epithelial cells and impair functioning of the mucociliary escalators, reducing the clearance of severe acute respiratory syndrome coronavirus (SARS-CoV-2). Obese diseased adipose tissues overexpress the receptors and proteases for the SARS-CoV-2 entry, implicating its possible roles as virus reservoir and accelerator reinforcing violent systemic inflammation and immune response. Finally, anti-inflammatory cytokines like anti-interleukin 6 and administration of mesenchymal stromal/stem cells may serve as potential immune modulatory therapies for supportively combating COVID-19. Obesity is conversely related to the development of COVID-19 through numerous molecular mechanisms and individuals with obesity belong to the COVID-19-susceptible population requiring more protective measures.

## 1. Introduction

The severe acute respiratory syndrome coronavirus (SARS-CoV) in Asia in 2002/2003 and the Middle East respiratory syndrome coronavirus (MERS-CoV) in the Middle East in 2012 caused severe respiratory diseases [1,2]. Currently, a novel SARS-like coronavirus 2 (SARS-CoV-2) is rapidly spreading over the world, resulting in the global coronavirus disease 2019 (COVID-19). COVID-19 causes high morbidity and mortality worldwide and the World Health Organization (WHO) officially declared this outbreak as pandemic [3]. Advancing progression of the infected lung leading to SARS is recognized as the most common complication of this disease. As of August 8, 2020, there have been 19,187,943 confirmed cases of COVID-19, including 716,075 deaths worldwide (WHO dashboard) [4]. The survival of severe COVID-19 might be followed by multiple long-term complications including lung insufficiency, vascular dysfunction, and heart failure. The COVID-19 pandemic poses a devastating challenge to the global health system and the economy.

The dramatic increase in the prevalence of obesity represents another urgent global challenge. Over the last decades, obesity has become a major epidemic in most industrialized countries, with extensive consequences for their health care systems [5]. This is exemplified by the fact that the percentage of children and adults with overweight has doubled, and even tripled in adolescents over the last three decades [6]. Obesity, commonly defined by the body mass index (BMI), is induced by an imbalance between food intake and energy expenditure [7]. Importantly, obesity is highly associated with a variety of disorders like cardiovascular diseases, insulin resistance, type 2 diabetes mellitus (T2DM), and various cancers [8,9]. These associations are likely connected to the hallmarks of obesity, which are characterized by a dysfunctional energy homeostasis promoting the development of the metabolic syndrome [10], diseased adipose tissues associated with local inflammation [11], hypoxia and cellular stress [11], altered release of cytokines [12], chronic systemic inflammation [13], insulin resistance [14], impaired immune response [15], atherosclerosis, and altered cardiac structure and function connected to a highly increased risk of cardiovascular diseases (CVD) [16]. Most of these hallmarks have direct or indirect negative impact on the well-balanced immune surveillance system leading to an impaired immune response, flawed chemotaxis, and deregulated immune cell differentiation [17]. Consequently, it is well documented that obesity is associated with an enhanced risk as well as worse course of infectious diseases with increased complications, prolonged hospitalization, and elevated critical illnesses [18,19,20].

The present coincidence of the COVID-19 and obesity pandemics exponentiates the aforementioned problems. Increasing evidence demonstrates that obesity is reversely associated with the development of COVID-19. In this review, we summarize the data of recent studies regarding the relationship between obesity and COVID-19 in terms of various clinical parameters and discuss the potential molecular linkages between these two pandemics, with a focus on a special cell population, namely, mesenchymal stem/stromal cells (MSCs).

## 2. Clinical Correlation of COVID-19 with Obesity

Studies concerning the H1N1 influenza A virus (H1N1) infection in 2009 identified obesity as a classified risk factor for infected patients for the first time, showing an increased severity of the disease and elevated mortality of patients [18]. This negative correlation between H1N1 infection and obesity was mainly associated with a delayed and reduced antiviral response towards the viral infection [18,21], poor effectiveness of antiviral drugs and vaccines [18,22,23], and boosted viral shedding/transmission as well as impaired clearance [18]. The correlation between obesity and COVID-19 was not clear at the beginning of this pandemic. Early COVID-19 studies, for example, from the “Chinese Center of Disease Control and Prevention” with 72,314 cases, did not provide sufficient patient data like body weight and height for calculating the BMI of patients [24]. This lack of data has changed recently, when multiple studies with large patient collectives were published from the UK [25], Mexico [26,27], and USA [28]. We have collected clinical reports (Table 1), which are supplemented with body weight, height, or the BMI, based on the publications in several online databases like PubMed, bioRxiv, Web of Science, etc. as of 24 July 2020.

Docherty and colleagues showed that obesity was significantly associated with a higher mortality (HR (Hazard Ratio): 1.33 (1.19–1.49, *p* < 0.001)) in a patient cohort of overall 20,133 cases [25]. This observation is supported by the New York study with 10,544 COVID-19 patients demonstrating that patients with a BMI of 30–40 kg/m^2^ had a significantly increased risk for hospitalization (HR: 4.26 (3.5–5.2, *p* < 0.001)) and clinical progression (HR: 1.38 (1.03–1.85, *p* = 0.029) compared to patients with a BMI below 30 kg/m^2^ [28]. Further support comes from a Mexican study with 4103 COVID-19 cases showing that patients with a BMI of ≥30 kg/m^2^ had a significantly reduced survival (HR: 1.74 (1.35–2.26, *p* < 0.001)) and increased hospitalization rate (HR: 1.64 (1.37–1.95, *p* < 0.001)) [26]. Another Mexican study with 51,633 patients showed similar results, with a significant increased lethality rate in patients with obesity (HR: 1.25 (1.17–1.34, *p* < 0.001) [27]. These reports with large patient cohorts are further strengthened by multiple clinical studies with smaller collectives worldwide [29,30,31,32,33,34,35,36,37,38,39,40,41,42,43,44]. Interestingly, Lighter et al. and Ebinger et al. showed that middle aged patients with an age below 52 and up to 60 years were more affected by obesity (BMI 30–34 and ≥35) with an HR: 2.0 (1.6–2.6, *p* < 0.0001)) and HR: 2.2 (1.7–2.9, *p* < 0.0001), respectively, resulting in increased morbidity rates compared to >60 years old patients [31,43]. Surprisingly, in the study by Ebinger and colleagues, obesity displayed the highest association with overall COVID-19 illness severity (*p* < 0.0001) correlated with patient age (<52 years vs. ≥52 years) of all comorbidities [43], further highlighting obesity as a high risk factor for middle aged adult patients. Moreover, three studies reported that poor prognosis of COVID-19 patients with obesity was gender-dependent, suggesting an association among obesity, male sex, and increased disease severity [29,35,45]. The study by Cai et al. reported that men with a BMI above 28 kg/m^2^ had a highly increased rate to progress to severe COVID-19 (HR: 5.4 (1.93–15.09, *p* = 0.006)), whereas the correlation was not so prominent in women (HR: 0.58 (0.07–5.24, *p* = 0.77)) [29]. This might be ascribed to the relatively low BMI of 28 kg/m^2^ in their collectives, while most of the other studies included patients with a BMI above 35 kg/m^2^. In contrast, the study by Hernández-Garduño suggested that women with obesity had a higher odds ratio for severe COVID-19 (HR: 5.5 (4.09–7.51, *p* < 0.0001) compared to men (HR: 4.72 (3.69–6.04, *p* < 0.0001) [35]. Interestingly, a rather small clinical study found that, in addition to the BMI, the area of visceral adipose tissue and upper abdominal circumference are important parameters for intensive care (HR: 22.53 (2.01–573.72) and intubation (HR: 16.11 (1.46–642.48) [46]. These studies strongly suggest a tight association between a high BMI/obesity and a severe course of the COVID-19 disease, particularly in middle aged men.

### Obesity-Related Comorbidities and COVID-19

Though also being present in obesity without comorbidities, this strong association is majorly observed in COVID-19 patients with obesity-related comorbidities. The increase in morbidity and mortality in patients with obesity is induced through various effects of obesity on nearly all systems of the human body [47]. It is even worse in infectious diseases like COVID-19. The four most prevalent comorbidities of COVID-19 are hypertension, diabetes, cardiovascular, and respiratory disease, all of them are tightly associated with obesity, and have a dramatic influence on the severity and mortality of COVID-19 [48,49]. The influence of obesity and its comorbidities was further assessed by a study reporting that obesity with diabetes, obesity with hypertension and diabetes, and diabetes with hypertension significantly increased the risk for hospitalization and mortality in a patient collective with 10,544 cases [26]. These data indicate that multiple obesity-associated comorbidities are significantly correlated to a worse prognosis suggestive of obesity being a high-risk factor for COVID-19 patients.

## 3. Adipose Tissue, Its Components, and Functions

Adipose tissue (AT) is a dynamic and crucial endocrine organ, secreting adipose tissue-derived hormones like adipokines/lipokines, endocrine factors, extracellular vesicles, enzymes, mRNAs, and microRNAs (miRNAs) modulating energy balance, glucose and lipid homeostasis, tissue repair, homeostasis, inflammatory, and immune response [11,50]. Especially, AT is capable of secreting a variety of cell signaling cytokines, known as adipokines [51], which regulate local and systemic inflammation as well as energy homeostasis [51]. Adipocytes in healthy AT are insulin-sensitive, a trait essential for adipocyte glucose uptake and for the modulation of hepatic gluconeogenesis, which enables the maintenance of normal blood glucose levels [52]. AT consists of lipid-filled mature adipocytes and several types of stromal cells including fibroblasts, adipose-derived mesenchymal stromal/stem cells (ASCs), endothelial cells, and various immune cells [53]. Interestingly, nearly all immune cells, such as resident macrophages, mast cells, monocytes, dendritic cells, natural killer cells, B cells, T cells, neutrophils, and eosinophils, have been found in AT [54].

### Adipose Tissue in Obesity

Obesity changes the composition, structure, and function of AT. Responding to excessive calorie intake, AT undergoes expansion via two processes: hyperplasia (increase in adipocyte number) and hypertrophy (increase in adipocytes size), and it is also influenced by endoplasmic reticulum stress, metabolic endotoxemia, or adipocyte death [55]. Expansion of adipocytes and insufficient vascularization lead to hypoxia; adipocyte apoptosis/necrosis; irregular fatty acid flux; and enhanced secretion of inflammatory adipokines, cytokines, and chemokines. This causes a massive immune cell infiltration that further promotes inflammation, stimulates lipolysis, and fuels insulin resistance, resulting in adipocyte dysfunction [56]. As a consequence, AT develops a local low-grade inflammatory microenvironment, which recruits inflammatory M1 macrophages, T cells, B cells, neutrophils, and mast cells. By contrast, the populations of T helper type 2 (Th2), M2 macrophages, and regulatory T cells (Treg) remain or even decrease in later stages of obese AT [11,57,58]. This shifts the balance from a regulatory anti-inflammatory immune state with the secretion of immunoregulatory cytokines including interleukin-4 (IL-4), IL-5, IL-10, IL-13, and IL-33 to a highly inflammatory state causing the secretion of monocyte chemoattractant protein-1 (MCP-1), tumor necrosis factor α (TNF-α), IL-1β, interferon γ (IFN-γ), and IL-6, leading to the development of a chronic and systemic inflammation [17].

Among proinflammatory cytokines, IL-1 family cytokines, as IL-1 and IL-18, are highly associated with obesity induced metabolic complications [59]. The production of these proinflammatory cytokines is mediated by NLRP3 (NOD-, LRR-, and pyrin domain-containing protein 3) inflammasome, which is crucial in immune responses, glucose homeostasis, lipid metabolism, and adipocyte functions. Multiple studies have reported that NLRP3 is activated in obese AT and associated with metabolic disorders [60]. Elevated inflammatory cytokines IL-6 and TNF-α are majorly produced by increased M1 macrophages in obese AT [61] and their altered circulating levels have been reported in patients with overweight and obesity [62], contributing to a local as well as systemic chronic inflammation. Further, the “trans-signaling” of the soluble form of the IL-6 receptor was shown to recruit macrophages into AT [63] and Mauer et al. highlighted the important role of IL-6 in the polarization of macrophages into the M2 subtype [64,65], both mechanisms are likely involved in the inflammatory maladaptation of obese AT.

This process is likely connected to a gradual loss of functional ASCs, a type of MSCs, in AT by reducing their differentiation, motility and immunomodulation capacity as well as impairing their ciliogenesis and pro-angiogenic ability [66]. Inflammation and its associated inflammatory cytokines, including IL-6, TNF-α, IL-1β, and inflammatory factors like C-reactive protein (CRP), are all known to induce endothelial dysfunction in AT [67]. Moreover, the deregulated expression of adipokines like leptin and resistin in AT of obese patients causes increased expression of vascular cell adhesion molecule 1 (VCAM-1) and intercellular adhesion molecule 1 (ICAM-1), both leading to vascular dysfunction and oxidative stress [68]. These factors contribute to dysfunctional/damaged endothelial cells and reduced angiogenesis worsening the hypoxic state of AT [69]. The hypoxic state induces the upregulation of hypoxia-inducible factor 1α that promotes fibrosis in an AT-specific manner by crosslinking collagen [57,70]. As a result, the extracellular microenvironment loses its flexibility, increases mechanical stress, restrains adipocyte expansion, and triggers adipocyte cell death and a sustained immune response in AT.

## 4. Obesity and COVID-19: Pathological Molecular Linkages

A great body of studies demonstrates a strong association between obesity, obesity-related comorbidities, and the severe outcomes of COVID-19. In the following, we discuss the issues that link obesity to the severe outcomes of COVID-19.

### 4.1. ASCs, MSCs, Obesity, and COVID-19

Among various cell types, ASCs are a specialized fundamental cell population in AT. These cells reside in human AT with multipotent differentiation features [71]. ASCs conduct their complex biological functions by direct cell–cell interaction and by indirect interaction through secretion of a variety of bioactive factors and microvesicles, exerting diverse functions including homeostasis, cell renewal, spontaneous repair, angiogenesis, immune modulation, and inflammation regulation [72,73,74]. Moreover, ASCs/MSCs have been shown to trigger changes in the energy metabolism, cell homeostasis, epithelial-to-mesenchymal transition, and fibrosis by stimulating various cellular pathways [75,76].

Interestingly, over the last two decades multiple investigations have revealed that morbid obesity compromises almost all ASC functions and properties, and changes their regulatory protective activity into a hypoxia and inflammation promoting phenotype [11,66]. In obesity, these cells lose their main features accompanied by a decrease in their multipotent state on account of reduced expression of pluripotency-associated genes like octamer-binding protein 4 (OCT4), SRY-Box transcription factor 2 (SOX2), homeobox transcription factor Nanog (NANOG), RNA exonuclease 1 homolog (REX1), and homeobox protein hox-C10 (HOXC10) [77,78]. Furthermore, ASCs/MSCs from obese patients are characterized by increased secretion of inflammatory cytokines including TNF-α, IL-8, IL-6, and MCP-1 [77,79]; reduced endothelial cell differentiation evinced by decreased secretion of vascular endothelial growth factor A (VEGF), hepatocyte growth factor (HGF), fibroblast growth factor 2 (FGF2), and platelet-derived growth factor (PDGF) [80,81]; and altered immune modulation capacity such as the inhibition of lymphocyte proliferation, recruitment of inflammatory monocytes and polarization of M1 macrophages [61,82], changed intracellular metabolism manifested by increased reactive oxygen species (ROS) production, impaired mitochondria function, and downregulation of sirtuin 1-6 (SIRT1-6) [11,83,84]. Moreover, as we reported, obesity impairs the ASC primary cilium, a unique signaling organelle on the cell surface, further contributing to their dysfunction in obese patients [66]. Interestingly, obesity-associated cytokines like IL-6 and TNF-α are able to influence many signaling pathways and induce defects in the ciliogenesis of ASCs [66,77,85]. Ciliary defective ASCs are not able to fulfill their physiological functions. Instead, these cells fuel inflammation in AT by secreting multiple inflammatory cytokines as IL-6 and IL-8 [66,85].

AT with its ASCs is present in various locations and in diverse organs. Of importance, ASCs are resident within the perivascular adipose tissue (PVAT) showing differentiation capacity towards adipocytes, endothelial cells, smooth muscle cells (SMCs), as well as osteoblasts for repair and replacement [86,87,88]. These cells also alter their functionality during the development of obesity, negatively affecting homeostasis and integrity of the endothelium, one of the major targets of SARS-CoV-2.

Moreover, patients with obesity may lose their functional ASCs in AT as well as MSCs in various organs including brain and lung [11,66]. Importantly, current clinical evidence suggests that pulmonary fibrosis or fibrous stripes is a severe complication observed in COVID-19 patients [89]. Pan and colleagues observed that 17% of hospitalized COVID-19 patients presented fibrous stripes [90]. Multiple studies demonstrate that loss or dysfunctional resident stem/MSC-like cells are responsible for the development of pulmonary fibrosis [91,92,93]. It has been revealed that resident lung perivascular ATP-binding cassette superfamily G member 2^+^ (ABCG2^+^) MSCs were decreased in human pulmonary fibrosis [93]. Moreover, perivascular glioma-associated oncogene^+^ (Gli1^+^) MSC-like cells, residing in various organs including lung, kidney, liver, and heart, generated myofibroblasts playing a central role in organ fibrosis after injury [92]. These data are underscored by Xie and colleagues reporting that transcription factor T-box gene 4 (TBX4)-lineage mesenchymal progenitors are the predominant source of myofibroblasts in injured adult lung [91]. In support of this notion, bleomycin-induced fibrosis is also associated with loss of resident lung MSCs [94]. In addition, Wnt/β-catenin signaling has been suggested to be an essential mechanism underlying the regulation of myofibroblast differentiation of lung-resident MSCs participated in the development of pulmonary fibrosis [95]. Thus, COVID-19 patients with obesity may suffer a more severe lung injury with pulmonary fibrosis owing to loss or dysfunctional lung resident MSCs induced by obesity. These MSCs in individuals with obesity may not be able to defend SARS-CoV-2 by modulating the immune response, tissue repair and homeostasis, and anti-inflammation. Conversely, obese ASCs/MSCs may further promote systemic inflammation and negatively impact the immune response [11].

Interestingly, ASCs/MSCs have been reported as cellular therapy option for pulmonary fibrosis and bronchopulmonary dysplasia by regulating immune cells inside the lung tissue, modulating trans-differentiation of different cell types including fibroblasts, and secreting various growth factors [96,97]. Furthermore, these cells could be used in infectious diseases because of their immunosuppressive or immune enhancement effects depending on their cytokine production and on the context of the immune response [98]. Many scientific and clinical trials are still ongoing to decipher how MSCs/ASCs regulate the immune system and several studies have shown their potential in regulating T cell proliferation or interacting with pathogens [99,100]. These cells even possess antimicrobial potential [101], highlighting the crucial roles of ASCs/MSCs in infectious diseases as well as their possible implications as novel cellular therapy for COVID-19 patients, especially for COVID-19 patients with obesity.

### 4.2. SARS-CoV-2 Receptors, Proteases, Adipose Tissue, and Obesity

SARS-CoV-2 uses its viral spike (S) protein for the entry into target cells. The spike protein consists of two functionally distinct subunits. The surface subunit S1 recognizes and binds to the cellular receptor, whereas the transmembrane subunit S2 facilitates fusion of the viral membrane with the cell membrane [102,103]. Like SARS-CoV, SARS-CoV-2 spike protein binds to its receptor human angiotensin-converting enzyme 2 (ACE2) through its receptor-binding domain (RBD) mediating its entry into host cells [104,105,106]. ACE2 is a zinc metalloprotease, which shares homology with ACE in its catalytic domain [107]. It has 805 amino acids including an N-terminal signal sequence and a C-terminal membrane binding domain [108]. ACE2 contains a single HEXXH zinc-binding motif and inactivates the potent vasoconstrictive peptide angiotensin II (Ang II) by removing its C-terminal phenylalanine residue to yield heptapeptide Ang-(1–7) [109]. The most remarkable expression of ACE2 protein was found on lung alveolar epithelial cells, and enterocytes of the small intestine, while ACE2 is present in arterial and venous endothelial cells, and arterial smooth muscle cells in all organs including oral and nasal mucosa, nasopharynx, stomach, colon, liver, kidney, and brain [110]. Moreover, based on bioinformatic and protein docking models, it has been suggested that, like MERS-CoV, the spike RBD of SARS-CoV-2 binds to human dipeptidyl peptidase 4 (DPP4) with a high affinity in addition to ACE2 [111,112]. Furthermore, cluster of differentiation (CD147)/basigin is recently proposed to be an alternative receptor for SARS-CoV-2 binding on the cell surface [113], although it is structurally not yet validated.

To fulfill its function, SARS-CoV-2 spike protein is proteolytically activated at its S1/S2 cleavage site by human transmembrane protease serine 2 (TMPRSS2) [104]. Apart from TMPRSS2, SARS-CoV-2 spike protein can be proteolytically activated by a variety of other proteases including furin, elastase, factor X, and trypsin, indicating the interesting fact that coronaviruses favor as receptors various protease proteins. These proteases are capable to perform a “priming” proteolysis that initiates the process of cellular entry [114,115,116]. Particularly, the spike protein of SARS-CoV-2 harbors a S1/S2 cleavage site containing multiple arginine residues, which is cleaved by the protease furin and is essential for spike protein mediated cell–cell fusion and entry into target cells [116]. SARS-CoV-2 depends on furin-mediated pre-cleavage of its spike protein at the S1/S2 site for subsequent spike protein activation by TMPRSS2 in lung cells [117].

AT expresses various receptors and enzymes required for SARS-CoV-2 infection. ACE2, the functional receptor for SARS-CoV and SARS-CoV-2, is highly expressed in AT [118,119]. Its mRNA was detected in human AT, with higher ACE2 expression in visceral compared to subcutaneous AT [120,121]. Importantly, its expression is upregulated in adipocytes of patients with obesity and diabetes [122]. In line with these observations, mouse AT and 3T3-L1 adipocytes display expression of ACE2 mRNA, protein, and enzymatic activity, and are regulated by high fat (HF) feeding [122]. Fatty acids confer multiple effects on gene expression, potentially related to activation of the peroxisome proliferator-activated receptor gamma (PPARγ) [123,124]. Interestingly, HF feeding induced an abundant expression of PPARγ in AT, which is associated with robustly increased ACE2 mRNA [122]. Obesity results in ACE2 upregulation in AT of mice causing mild epicardial AT inflammation [125]. Recently, a study with 5457 COVID-19 patients showed that individuals with obesity demonstrate significantly higher levels of ACE2 in their blood sera [126]. In sum, ACE2 is highly expressed in adipocytes and AT, and its expression is increased in obesity, which could turn AT into a potential target and viral reservoir, as suggested [127].

Other suggested receptors for SARS-CoV-2 are also present in AT. DPP4, the potential SARS-CoV-2 receptor, is multifunctional including its roles in glucose homeostasis, inflammation, and the immune system [128]. Identified as a novel adipokine in AT [129], DPP4 is strongly expressed on the apical surfaces of the polarized epithelium of various organs such as lung and liver, and increased DPP4 results in failures to resolve inflammation and chronic subclinical activation of the immune system [128]. Interestingly, DPP4 is upregulated in obesity, especially in the insulin resistance state [129,130]. Inhibition of DPP4 prevented fibrosis in obese white AT [131]. AT, and specifically adipocytes, have been proposed to be a significant circulating source of DPP4 [132]. DPP4 secretion from AT was also demonstrated in vivo with greater release in obese compared to lean individuals [133]. Thus, AT from obese patients highly expresses DPP4 and possibly is its major circulating source, which may facilitate the entry of SARS-CoV-2 into cells and also strong inflammation and violent immune response, important steps leading to the cytokine storm of COVID-19. It will be of high interest to decipher the roles of DPP4 in mediating SARS-CoV-2 entry as well as in inflammation and immune response in COVID-19 patients with or without obesity. Interestingly, DPP4 inhibitors were introduced as 2nd to 4th-line treatment option of type 2 diabetes [134], and they might be a suitable combinatory treatment option for COVID-19 patients. In addition, the expression of CD147, the suggested alternative receptor for SARS-CoV-2 [113], is positively correlated with the BMI possibly contributing to the COVID-19 morbidity and severity patterns [135].

Moreover, the protease TMPRSS2 is expressed in AT, though at a low level [136], while furin is enhanced expressed in obese AT as well as during adipogenesis [137]. Furin supports not only the entry of SARS-CoV-2 into cells, but also the exit of virus particles from cells by priming the spike protein [138]. New viral particles can attack the neighboring cells or be released into the circulation.

**Figure 1:**
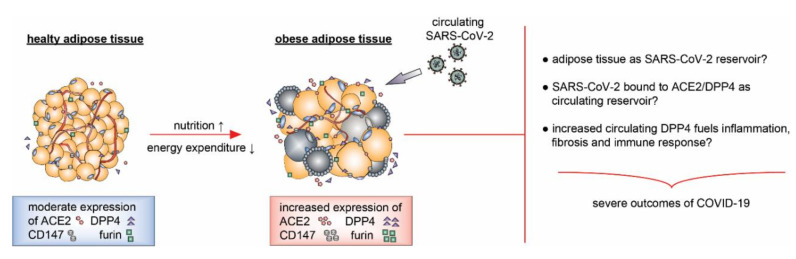
The illustration indicates that adipose tissue expresses the receptors ACE2, DPP4, and CD147, and the protease furin for the SARS-CoV-2 entry. These proteins are upregulated in obese adipose tissues accompanied by an enhanced secretion of ACE2 and DPP4 in the circulation of obese patients. Diseased adipose tissues could be targeted by SARS-CoV-2 and serve as its reservoir, as well as an accelerator reinforcing systemic inflammation and immune response, resulting in severe outcome of COVID-19 in obese patients.

As AT contains almost all components for SARS-CoV-2 entry into and exit from cells, and some of these components are highly involved in inflammation and immune response, it is tempting to suggest that AT, especially AT of obese patients, could serve as a SARS-CoV-2 target organ as well as its viral reservoir [127]. This reservoir could act as an accelerator reinforcing vigorous inflammation, fueling a storming immune response, damaging tissues, and causing multi-organ failure, representing severe complications of COVID-19 (Figure 1). Experimental investigations are required to examine these hypotheses.

### 4.3. Obesity-Related Inflammation and Immune Responses

AT is predominantly characterized by inflammatory and dysfunctional immune response in patients with morbid obesity. The extensive intake of nutrients and saturated free fatty acids has been shown to stimulate toll-like receptors (TLRs) expression in dendritic cells (DCs) [139], especially TLR2 and TLR4, known for their role in activating M1 macrophages [140]. Second, increased levels of lipopolysaccharide and adenosine triphosphate activate the NLRP3 inflammasome, mediating reactive oxygen species (ROS) activation and the release of IL-1β and IL-18 [141]. Third, the activation of M1 macrophages outside of AT is further enhanced by increased secretion of AT inflammatory cytokines TNF-α, IL-6, visfatin, resistin, angiotensin II, and plasminogen activator 1 into the blood [142]. Fourth, the secretion of important adipocytokines in particular leptin impacts the immune response by augmenting the production of TNF-α, IL-6, and IL-12, leading to a predominantly pathogenic proinflammatory T helper type 1 cell (TH1) population [143]. Crucial downstream targets of these cytokines are inhibitor of nuclear factor Kappa-B kinase subunit beta (IKKβ), nuclear factor Kappa B subunit 1 (NF-κB), and mitogen-activated protein kinase 8 (JNK), triggering endoplasmic reticulum (ER) stress associated with the activation of the unfolded protein response pathway [144]. Fifth, the regulated expression of miRNAs is important in immunomodulation and more than 23 circulating miRNAs were deregulated in obese patients [145]. These miRNAs have implications in immune cell development, T lymphocyte generation, lipid metabolism, and macrophage proinflammatory responses [144]. In conclusion, multiple obesity-associated factors have tremendous impact on the immune response by activating the proinflammatory cell populations of Th1 cells, M1 macrophages, CD8^+^ T cells, and DCs.

In accordance with an impaired immune response, several clinical and animal studies showed that obesity increased the severity and mortality rate of individuals with obesity in infectious diseases like influenza [18,146]. Obese mice induced either by high fat diet or leptin receptor deficiency had a highly impaired immune response to influenza virus. Obese mice suffered from decreased numbers of bone marrow-resident B cells, increased cytotoxic CD8^+^ T cells, and reduced Treg cells, resulting in increased inflammation and damage in the lung, a reduced response to adjuvant vaccination, and reduced virus clearance [147,148,149]. Additionally, obese mice had altered metabolic profiles after infection with H1N1 in several organs including lung, liver, AT and blood [150], suggesting a relationship between impaired tissue metabolism, elevated infection, and mortality rates in obese individuals. In support of these observations, obese patients had a significant reduced influenza specific antibody titer one year after postvaccination [151,152]. Furthermore, the peripheral blood mononuclear cells from influenza vaccinated adults with obesity had less active CD4^+^ and CD8^+^ T cells with reduced marker expression of CD28, CD40 ligand, CD69, IL-12R, and IFN-γ. In line with these findings, another study showed that vaccinated adults with obesity had twice the risk of influenza or influenza-like illnesses compared to healthy lean adults [23]. This indicates that obesity diminishes the immune response observed in healthy adults even after vaccination [23], which should be taken into consideration for potential SARS-CoV-2 vaccines in the future.

### 4.4. Obesity and Endothelial Dysfunction

The endothelium regulates vascular homeostasis by maintaining a delicate balance between the secretion of vasodilators and vasoconstrictors. It produces a series of bioactive mediators that moderate vascular tone, control permeability, modulate proliferation, regulate migration of smooth muscle cells, decrease leukocyte migration, and regulate platelet adhesion and aggregation [67]. Obesity-associated inflammation causes an imbalance between proinflammatory/pro-coagulant and anti-inflammatory/anti-coagulant states of the endothelium, thus, contributes to its disturbed hemostasis. Various studies reveal that endothelial dysfunctions in obesity develop from a chronic and progressive inflammatory process [153]. Diverse cell types like adipocytes, ASCs, and immune cells in obesity secrete and release various proinflammatory factors including IL-6, IL-1, TNF-α, leptin, and MCP-1. These cytokines activate endothelial cells by enhancing leukocyte and monocyte adhesion to the endothelium and inducing infiltration of proinflammatory macrophages, which in turn increase levels of inflammatory factors, worsening inflammation in the endothelium [154]. In particular, PVAT plays a crucial role in obesity-induced vascular dysfunction. Hypoxia, inflammation, and oxidative stress in PVAT lead to an impairment in the release of vasoactive factors from PVAT, and the normal anti-contractile function of PVAT is lost in obesity [155].

Increased inflammation and dysregulated metabolic processes have a profound influence on endothelial dysfunction associated with the formation of atherosclerotic plaque [67]. Interestingly, all cells in atherosclerotic plagues, like endothelial cells, monocytes/macrophages, and smooth muscle cells, express furin [156], one of the priming proteases for SARS-CoV-2. Furin expression levels correlate with atherogenesis [157]. Recently, it has been revealed that expressed furin in vascular endothelial cells promotes the NF-κB activity; the expression of VCAM-1, MCP-1, and monocyte–endothelial adhesion; and transmigration [158], which results in further inflammation and damage of vascular endothelium. In addition to furin, vascular endothelium also highly expresses the ACE2 receptor [110] and the priming protease TMPRSS2 [159], which make the endothelium of patients with obesity highly susceptible to SARS-CoV-2 infection. The viral infection results in increased cell death of endothelial cells, triggering an enhanced release of proinflammatory mediators and increased recruitment of inflammatory/immune cells. In fact, a recent study based on post-mortem analyses of COVID-19 patients demonstrated viral inclusion bodies in endothelial cells, accumulation of inflammatory cells on the endothelium, congestion of the small lung vessels, and endotheliitis of the submucosal vessels of the small intestines in patients who died of COVID-19 [160]. The authors further proposed that induction of apoptosis and pyroptosis might have an important role in endothelial cell injury in COVID-19 patients [160]. Obesity diseased endothelium may facilitate SARS-CoV-2 infection and cause widespread endotheliitis, coagulopathy, arterial and venous thromboses.

Moreover, obesity-related inflammation and metabolic dysregulation change the whole landscape of the endothelium accompanied by a variety of structural, functional, and molecular alterations including alteration in nitric oxide (NO) and ROS. NO produced by endothelial NOS (eNOS) relaxes vascular smooth muscle cells; prevents their excessive proliferation; increases blood flow, suppresses platelet aggregation; inhibits the activation of endothelial cells; and suppresses the release of mediators that recruit leukocytes, monocytes, and macrophages to the endothelium [161,162]. Unfortunately, endothelial NO concentration and production are suppressed in obesity [163,164]. Instead, inducible NOS (iNOS) produces much higher and toxic levels of NO and is found in adipocytes and proinflammatory macrophages. NO production by iNOS is elevated in obesity [165]. Moreover, ROS are often greatly elevated in obesity and causes serious pathological alterations. Chronic hypernutrition induces the production of superoxide (O_2_^-^), mitochondrial oxidative phosphorylation, and endothelial dysfunction/eNOS uncoupling [166,167,168]. Chronic inflammation in obese AT can further promote the infiltration of inflammatory ROS-producing macrophages [169,170].

Collectively, obesity disrupts the delicate balance and promotes the development of vascular endothelial dysfunction. Importantly, vascular endothelium in patients with obesity may highly express ACE2, TMPRSS2, and furin, which make it more vulnerable to SARS-CoV-2 infection, impairing this balance. Alterations in this balance predispose the vascular endothelium toward pro-thrombotic and pro-atherogenic states, resulting in platelet activation, impaired coagulation, and thrombosis, leading subsequently to damage and failure of vital organs [67,171]. Endothelial dysfunction presented in obesity significantly contributes to obesity-related comorbidities, such as hypertension, diabetes, and dyslipidemia.

## 5. Obesity and Its Comorbidities

The development of obesity has a multifactorial etiology including individual genetics and epigenetics, social and family environment, the nervous system, and psychological and metabolic factors [172]. The resulting disequilibrium between energy intake and expenditure causes a disproportional accumulation of AT [173]. This excessive expansion of AT leads to the formation of class III obesity (morbid obesity, BMI > 40 kg/m^2^), which is associated with at least 18 comorbidities highlighted in a comprehensive meta-analysis report [174]. These studies indicate that obesity is significantly correlated with T2DM and bears an increased risk for several cancer entities, hypertension, coronary heart disease, congestive heart failure, pulmonary embolism, stroke, and asthma [172,174], resulting in higher mortality rates in individuals with morbid obesity [175]. As this diseased AT of obese patients has various implications in nearly every system of the human body [176], it is not surprising that the most impactful comorbidities of COVID-19 are in direct relation to obesity.

### 5.1. Obesity, Diabetes, and Their Implications in COVID-19

AT is an important contributor to the insulin sensitivity, glucose homeostasis, and dyslipidemia by regulating lipid metabolism, glucose uptake, and endocrine regulation [177]. Obesity combined with insulin resistance often leads to the development of T2DM, one of the serious comorbidities for COVID-19 patients [28,178]. The molecular mechanisms behind the insulin resistance are dependent on various obesity related factors. The first measurable change after extensive energy intake, for example, after HF feeding in animal models, is an elevated level of circulating insulin that develops into a hyperinsulinemia at later stages [179,180]. This is accompanied by a hyperglycemia at least partly induced by the upregulation of forkhead box O1 (FOXO1) in hepatocytes, increasing the expression of gluconeogenesis-related proteins and the impaired translocation of GLUT4 in muscle cells [181]. Additionally, the response to insulin is also defective in AT from obese individuals, induced by high levels of TNF-α that interfere with the insulin signaling transduction by activating IKK and MAPK, both of which perform inhibitory phosphorylation on insulin receptor substrate 1 and 2 [182]. AT from obese patients produces high levels of retinol-binding protein-4 (RBP4), an antagonist of the phosphoinositid-3-kinasen (PI3K), resulting in a deregulated expression of gluconeogenic enzyme phosphoenolpyruvate carboxykinase associated with increased triglyceride levels in the serum and fat deposition in the liver [183]. Moreover, the secretion of proinflammatory cytokines like TNF-α, IL-6, and MCP-1 promotes JNK, IKK-β/NF-κB, and inducible iNOS pathways, further enhancing the inflammatory process induced by obesity, facilitating the development of insulin resistance [14,184]. In line with this, omental adipose tissue of insulin-resistant patients has a significant increased infiltration of macrophages compared to insulin-sensitive patients, further enhancing the inflammation signaling inside the AT [185]. Furthermore, the release of high levels of non-esterified fatty acids (NEFA) could be a key component by linking extensive adipose tissue mass to insulin resistance, β-cells dysfunction, and apoptosis [186]. This is ascribed to highly increased plasma level of NEFA, also connected to lipotoxicity, decreased insulin release from β-cells, increased production of metabolites such as diacylglycerol (DAG), fatty acyl-coenzyme A (ACLS) interfering with the downstream activation of insulin receptor signaling [186,187]. The development of insulin resistance is characterized by hyperglycemia, hyperlipidemia, and hyperinsulinemia [188], indicating that diabetes is tightly associated with the hallmarks and progression of obesity.

The coherence between obesity, diabetes, and COVID-19 is suggested at least by five different factors: viral load, immune response, alveolar dysfunction, endothelial dysfunction, and coagulopathy [189]. As ACE2 mediates the entry of SARS-CoV-2 into the host cells, and an increased expression of ACE2 was detected in lung, kidney, and heart tissue of diabetic mice [190], diabetes could foster the viral load in COVID-19 patients. Next, the immune response is altered in patients with obesity and diabetes, both factors are associated with a chronic low-level inflammation [11,191], reduced NK cell activity, and deregulated numbers of CD4^+^ and CD8^+^ T cells [189], which could cause a delayed immune response and a prolonged hyperinflammation in COVID-19 patients. Moreover, molecular mechanisms of impaired endothelium in patients with diabetes are complex and connected to oxidative stress, inflammation, and a change in the hemodynamic balance [82]. Increased expression of eNOS, elevated production of ROS, activation of NF-ĸB, and chronic inflammation are some of the key factors for endothelial dysfunction with a shift to a vasoconstrictor, pro-thrombotic and chronic inflammatory state [171,192], likely fueling the severe impairment of COVID-19 on the cardiovascular system. Finally, severely ill COVID-19 patients are often associated with coagulopathy/thrombosis [193] and obesity as well as diabetes are characterized by a hypercoagulable state [194]. In obesity, this symptom is associated with the overexpression of multiple proteins associated to coagulation like thrombin, fibrinogen, coagulation factors (FXa, FVIIa, and FV), and activated protein C in diverse cell types including adipocytes, endothelial cells, and vascular smooth muscle cells [194]. This is even enhanced in patients with obesity and diabetes by acute hyperglycemia and hyperinsulinemia that were shown to trigger coagulation and fibrinolytic activity during inflammatory response [189]. Consequentially, patients with obesity and diabetes are likely more affected by coagulopathy, but reliable data correlating these factors are still missing.

### 5.2. Obesity and Its Connection to Hypertension and Respiratory Diseases

Hypertension, one of the most prominent comorbidities of patients with obesity, is induced by multiple mechanisms. As aforementioned, damaged endothelium as well as systemic inflammation in obesity favor the formation of hypertension [195]. Moreover, patients with morbid obesity have higher plasma concentrations of renin, angiotensinogen, ACE, Ang II, and aldosterone, activating the renin–angiotensin–aldosterone system (RAAS). Increased natrium chloride reabsorption and blood pressure as well as reduced arteriolar resistance are all known factors in the development of hypertension and cardiovascular diseases in obesity [196,197].

**Figure 2:**
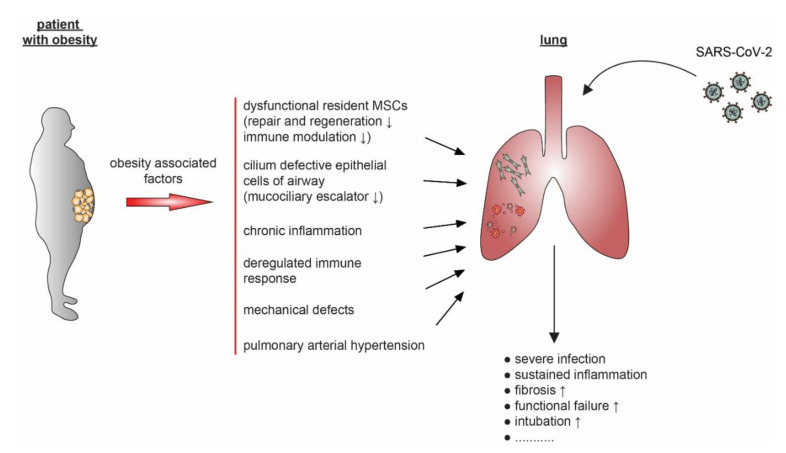
A model showing negative effects of obesity on the pulmonary pathogenesis of COVID-19. Obesity-associated aspects, including defective immune response, chronic inflammation, dysfunctional MSCs, compromised ciliated airway epithelial cells, mechanical defects, and pulmonary arterial hypertension, impair the lung defense system against SARS-CoV-2 infection and cause worse outcome of obese COVID-19 patients.

Negative impacts of obesity on lung function are versatile and weaken the lung defense against SARS-CoV-2 (Figure 2). The implication of obesity in respiratory diseases can initially be ascribed to two pathomechanisms: the mechanical/physiological and the inflammatory/metabolic part [198]. Many studies over the last decades show that disproportional weight gain accompanied with fat deposition on the chest wall, abdomen, and upper airway are associated with decreased lung volume, forced expiratory volume in 1 second (FEV1), forced vital capacity (FVC), functional residual capacity (FRC), and expiratory reserve volume (ERV) [199]. The second factor is again associated with systemic inflammation triggered by obesity. The increased levels of adipokines including leptin, resistin, and visfatin; proinflammatory cytokines/chemokines like MCP-1, IL-6, IL-8, and TNF-α; and other factors such as free fatty acids and triacylglycerol increase the vulnerability of the lung to pulmonary inflammation [200]. This has implications in enhanced leukocyte recruitment, cytokine production, microvascular permeability, airway obstruction, and damaged endothelial cells [200] causing a highly increased risk for several respiratory systemic diseases [201]. Third, in combination with systemic inflammation, insulin resistance, and oxidative stress, expansion of AT surrounding the pulmonary artery contributes to the pathobiology of pulmonary arterial hypertension [202], impairing the structure and function of the lung. Fourth, as discussed, patients with obesity may lose their functional lung MSCs, vulnerable to the development of pulmonary fibrosis, a severe complication observed in COVID-19 patients [89]. Fifth, obesity may compromise motile cilia on airway epithelial cells and impair functioning of the mucociliary escalators, which could render difficulty in clearing the invading SARS-CoV-2. Motile cilia are traditionally considered to work as mechanical machines responsible for clearing solutes, debris, and/or pathogens, whereas primary cilia are regarded to modulate sensing and signaling environment [203]. However, studies demonstrate that, like primary cilia, motile cilia have various receptors, and are capable of sensing and regulating signal transduction in ciliated cells [204]. As primary cilia on a diversity of cells are negatively affected by obesity [66], it is tempting to speculate that obesity deregulates motile cilia on airway epithelial cells as well. It will be of importance to compare the morphology and function of motile cilia on airway epithelial cells between lean and obese individuals, and to examine potential alterations after the exposure to coronaviruses like SARS-CoV-2.

## 6. Obesity and Cytokine Storm in COVID-19

The severe manifestation of COVID-19 is characterized by an uncontrolled extensive production of soluble inflammatory cytokines, an aberrant systemic inflammatory response, and associated very often with the acute respiratory distress syndrome (ARDS) that has been described in up to 20% of COVID-19 patients [205]. This so-called cytokine storm is a commonly induced complication by viral infections and its incidence rate averages between 3.7% and 4.3% in all sepsis cases [206]. It was found to be the major cause of morbidity in patients infected with SARS-CoV and MERS-CoV with elevated IL-6 and other cytokines [2,207]. Cytokine storm is characterized by highly increased levels of IL-6, TNF-α, G-CSF (granulocyte colony-stimulating factor), IP10 (interferon-γ inducible protein 10, also known as CXCL10), MCP1, MIP1-α (macrophage inflammatory protein 1-α), IL-2, and IL-7 in patient blood [206,208,209]. SARS-CoV-2 may infect monocytes, macrophages, and dendritic cells resulting in their activation and secretion of IL-6 and other inflammatory cytokines [210]. On the other hand, patients with obesity already suffer from chronic inflammation accompanied by elevated inflammatory cytokines, suggesting that these individuals with increased inflammatory cytokines and dysfunctional immune response are more susceptible to this severe complication, though conclusive clinical data are still being awaited. Clinical studies reported an increased severity of patients with obesity, which are infected with the H1N1 virus [211]. Moreover, an animal study reported that, compared to lean mice, obese mice developed a more intense reaction to proinflammatory cytokines associated with a cytokine storm and increased morbidity [212]. These phenomena could be presumably true for COVID-19 patients with obesity, which would make an intense monitoring and therapeutic intervention mandatory for these patients.

## 7. Anti-Obesity-Related Therapies: Potential Strategy for Combating COVID-19

No specific drugs or vaccines are currently available to cure patients with COVID-19. Along with the development of antivirals and vaccines that specifically prevent or ameliorate the SARS-CoV-2 infection, several anti-obesity-related aspects could be considered as a supportive therapy, for example, anti-inflammatory cytokines like anti-IL-6 and administration of MSCs.

The crucial involvement of IL-6 in COVID-19 is strengthened by a recent report that impaired immune cell cytotoxicity in severe COVID-19 is IL-6-dependent [213], underlining the importance of anti-IL-6 in combating severe COVID-19. Interestingly, IL-6 receptor antagonists have been shown to reduce the mortality of patients with a cytokine release syndrome induced by chimeric antigen receptor T cell therapy leading to the FDA approval of such therapies [214]. In fact, multiple studies using tocilizumab, a recombinant humanized anti-human IL-6 receptor monoclonal antibody that binds both membrane bound IL-6 receptor (mIL6R) and soluble IL-6 receptor (sIL6R), have been carried out to treat severely ill COVID-19 patients, as recently summarized [215]. Encouraging results were initially reported on small case series of severe COVID-19 patients in China [216,217]. However, the results from a recent study with a large cohort of severe COVID-19 patients treated in an off-label access with tocilizumab were not satisfying [218]. Randomized controlled trials will clarify the efficacy of tocilizumab and the best timing of its administration in different patient categories. Alternatively, anti-IL-6 agents majorly targeting sIL6R (IL-6 trans-signaling, endothelial cells) but less targeting mIL6R (IL-6 cis-signaling, lymphocytes) should be administrated like Olamkicept/glycoprotein 130, which is already used in phase II clinical trials [219], so that immune cells are less targeted reducing severe immunosuppressive side effects as observed with tocilizumab. Moreover, as IL-6 works through the Janus kinase–signal transducer and activator of transcription (JAK/STAT) pathway [219], selectively targeting this pathway will be useful to regulate the IL-6 activity. To modulate, but not completely block, the activity of IL-6 may open a new path to treat severely ill COVID-19 patients by reduced complications.

Second, the administration of MSCs could be considered to modulate dysfunctional immune response and to regenerate damaged tissues induced by SARS-CoV-2. Obesity-associated factors compromise functionalities of MSCs in various organs as well as in AT, and MSC restoration is useful for tissue regeneration, anti-inflammation, and immune modulation [11,66]. There are several studies with small case numbers of COVID-19 patients treated with MSCs derived from the umbilical cord (UC-MSCs) [220,221]. Encouragingly, Leng and colleagues reported that in the UC-MSCs treated group, the clinical parameters, including the oxygen saturation, inflammation and tissue injury biomarkers, and aspartic aminotransferase, were improved or normalized [220]. Moreover, a positive clinical outcome was also demonstrated in a severe COVID-19 patient treated with intravenous infusion of human umbilical cord Wharton’s Jelly-derived MSCs [222]. These limited clinical data imply that MSCs exert their anti-inflammatory and immunomodulatory actions in COVID-19 patients. Several clinical trials with lager collectives are ongoing, which hopefully provide more reliable data. MSCs, especially UC-MSCs, might be useful for preventing and minimizing cytokine storm in the acute phase of COVID-19 as well as for repairing and regenerating damaged tissues in the recovery phase of severe COVID-19 survivors, especially in patients with obesity.

## 8. Conclusions

**Figure 3:**
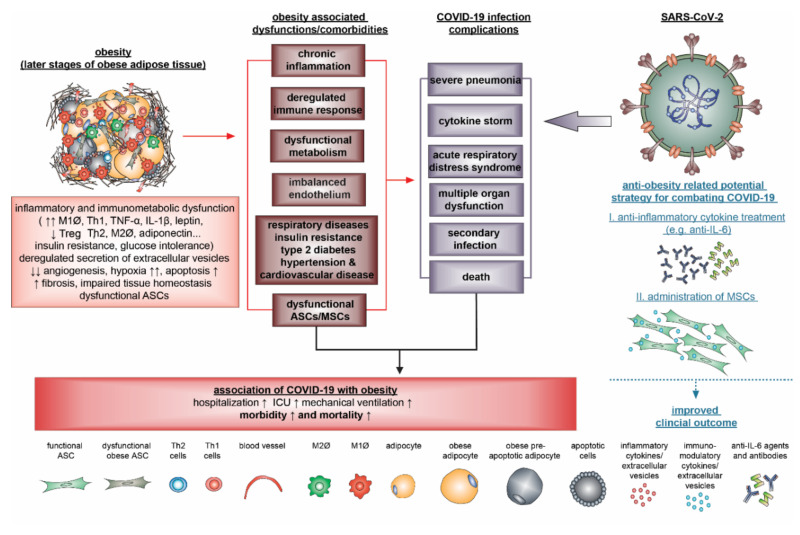
Schematic illustration presenting that obesity negatively impacts the development of COVID-19. Obesity is characterized by various pathological features, including systemic chronic inflammation, deregulated immune response, dysfunctional endothelium, increased comorbidities, and dysfunctional ASCs/MSCs, which fundamentally influence the progression and outcome of COVID-19. Anti-inflammatory cytokine therapies for example anti-IL-6 and administration of MSCs may be useful for supportively treating COVID-19.

Multiple studies reveal a strong association between COVID-19 and obesity. COVID-19 patients with obesity have an enhanced hospitalization rate, more severe progression, and worse clinical outcomes. In particular, cytokine storm, a severe complication of COVID-19, could be associated with obesity, though conclusive clinical data are still being awaited. Apart from obesity-related comorbidities, systemic chronic inflammation, deregulated metabolism, dysfunctional immune system, inflamed endothelium, impaired MSCs, and altered AT play crucial roles in bridging obesity to critically severe outcomes of COVID-19 (Figure 3).

In particular, loss or dysfunctional lung resident MSCs may cause more severe lung injury with pulmonary fibrosis in COVID-19 patients with obesity. Obesity may also compromise motile cilia on airway epithelial cells and impair the function of the mucociliary escalators responsible for clearing SARS-CoV-2. Obese diseased adipose tissue that contain all components for SARS-CoV-2 infection could be targeted and even serve as a virus reservoir, and an accelerator reinforcing violent systemic inflammation and immune response, facilitating the development of a cytokine storm, a severe complication of COVID-19. This highlights the key role of inflammation and the cytokine network in COVID-19 patients with obesity. Individuals with obesity are highly susceptible to SARS-CoV-2 infection and more protective measures should be taken for this population. Anti-inflammatory cytokine therapy like anti-IL-6 and administration of MSCs may have potential for supportively treating COVID-19.

## Figures and Tables

**Table 1:** Clinical reports of COVID-19 patients with obesity.

Case Number	BMI (kg/m^2^)	Hazard Ratio (HR)/ *p*-Value	Country/City	Clinical Relevance	Author/Year
**20,133** **14,396** **1685**	lean obese	1.33 (1.19–1.49, *p* < 0.001)	UK/multicenter	Obesity increased significantly the risk for hospitalization and mortality.	Docherty et al. 2020
**3003** **915** **185**	<30 30–40 >40	1.38 (1.03–1.85) 0.029 1.73 (1.03–2.90) 0.038	USA/New York	Strong associations of obesity with hospitalization. Obesity had the strongest association with critical illness and a substantially higher OR than any cardiovascular or pulmonary disease.	Petrilli et al. 2020
**51,633** **40,925** **10,708**	lean obese	Lethality: 1.25 (1.17–1.34, *p* < 0.001)	Mexico/Mexican	Obesity raises the risk of an infection with COVID-19. It increases mortality and diseases severity.	Bello-Chavolla et al. 2020
**124**	25–30 30–35 ≥35	1.69 (0.52–5.48) 0.22 3.45 (0.83–14.31) 0.48 7.36 (1.63–33.14) 0.021	France/Lille	High frequency of obesity among patients admitted in intensive care. Disease severity increased with BMI. Obesity was a risk factor for COVID-19 severity. Patients with obesity required more mechanical ventilation (BMI ≥ 35 at 85.7%).	Simonnet et al. 2020
**141** **99** **173** **134**	age ≥ 60 years 30–34 ≥35 age < 60 years 30–34 ≥35	0.9 (0.6–1.2) 0.39 0.9 (0.6–1.3) 0.59 2.0 (1.6–2.6) < 0.0001 2.2 (1.7–2.9) < 0.0001	USA/New York	Obesity was a risk factor for hospital admission and patients with obesity needed more critical care. Obesity in people < 60 years is a newly identified epidemiologic risk factor, which may contribute to increased morbidity rates documented in USA.	Lighter et al. 2020
**102**	24.4 (all) 26.0 (non-survivors) 24.3 (survivors)	0.088	China/Wuhan	Deceased patients had a slightly yet not significant increased BMI compared to survived patients.	Cao et al. 2020
**383** **203** **123** **41**	18.5–23.9 24.0–27.9 ≥28	1.86 (1.00–3.46) 0.05 3.42 (1.42–8.27) 0.006	China/Shenzhen	Obesity, especially in men, significantly increased the risk for developing severe pneumonia in COVID-19 patients.	Cai et al. 2020
**422** **71**	≥30	2.04 (1.14–3.65) 0.016 association with age: <0.001	USA/ Los Angeles	Obesity and diabetes mellitus were associated with greater OR of needing hospitalization and increased risk of pneumonia. Obesity, diabetes, or an elevated overall comorbidity index were individually associated with illness severity in younger COVID-19 patients (i.e., <52 years).	Ebinger et al. 2020
**-**	≥30	*p* < 0.001	140 countries included	People above 65 years of age, obesity, and urbanization were all positively associated with COVID-19 mortality.	Squalli 2020
**10,544** **2097**	≥30	hospitalized: 1.64 (1.37–1.95) (*p* < 0.001) survived: 1.740 (1.35–2.26) (*p* < 0.001)	Mexico/ Mexico City	Hypertension, obesity, and diabetes presented in combination, provided a higher risk of hospitalization and mortality in comparison with patients without these comorbidities.	Carrilo-Vega et al. 2020
**112** **79** **33**	< 25 ≥25	mortality: *p* < 0.001	China/Wuhan	Obesity correlated with increased mortality in COVID-19 patients.	Peng et al. 2020
**1158** **266** **98** **40** **19**	< 25 30–34.9 35–39.9 >40	BMI > 40: 3.95 (1.00–15.20, *p* = 0.046)	Kuwait/Kuwait city	Overweight, obesity and diabetes were associated with intensive care and poor outcomes of patients with COVID-19.	Al-Sabah et al. 2020
**200** **38** **116** **46**	<25 24–34 ≥35	mortality: 2.56 (1.18–5.57, *p* = 0.018) oxygen: 2.16 (1.08–4.34, *p* = 0.030) intubation: 2.72 (1.24–5.96, *p* = 0.012)	USA/New York	COVID-19 patients with obesity had an increased risk of in-hospital mortality, oxygen requirement and intubation.	Palaiodimos et al. 2020
**30**		ICU: 22.1 (2.17–486.27) intubation: 13.92 (1.56–379.04)	Germany/Berlin	Visceral adipose tissue and upper abdominal circumference specifically increased the risk of COVID-19 severity. An increase in visceral fat area by 1 dm^2^ was associated with a 22.53-fold increased risk for ICU treatment and a 16.11-fold increased risk for mechanical ventilation.	Petersen et al. 2020
**172** **148** **24**	<27 >28	severe illness: 6.90 (2.38–19.97, *p* < 0.001)	China/Jiangsu	The BMI of COVID-19 patients was independently correlated with severe illness and increased intensive care treatment.	Huang et al. 2020
**124** **30** **59** **35**	<25 >30 <30	ICU: 7.36 (1.63–33.14, *p* = 0.02)	Germany/ Georgsmarienhütte	A clear correlation between the BMI of COVID-19 patients and the likelihood for ICU and a worse disease progression.	Müssig 2020
**12,269** **7552** **4717**	<35 >35	1.31 (1.25–1.37) *p* < 0.001	Mexico/Mexico City	Obesity is suggested as the strongest associated comorbidity for COVID-19. Comparing the odds ratio between male and female displays an increased risk for females with obesity for COVID-19.	Hernández-Garduño 2020
**172** **155** **17**	<25 >30	*p* = 0.002	Spain/Ciudad Real	Patients suffering from obesity have a highly increased risk of ICU requirement.	Urrra et al. 2020
**770** **28** **465** **277**	<18.5 18.5–30 >30	ICU: *p* = 0.001 intubation: *p* < 0.001 death: *p* < 0.001	USA/New York	The disease severity and critical care requirements are increased in COVID-19 patients with obesity. This is associated with augmented rates of ICU admission and increased mortality.	Hajifathalian et al. 2020
**92** **32** **31** **29**	22.3 ± 1.9 27.4 ± 1.5 32.4 ± 2.6	hospitalized: *p* < 0.001 ventilation: 4.19 (1.36–12.89, *p* = 0.012) ICU: 11.65 (3.88–34.96, *p* < 0.001)	Italy/Veneto	Patients with overweight or obesity have an increased rate of hospitalization combined with a related pneumonia. They required more frequently non-invasive mechanic ventilation and invasive mechanic ventilation associated with an elevated rate of ICU requirement.	Busetto et al. 2020
**387** **99** **41**	<29.9 >30	acute respiratory distress *p* < 0.001	Italy/Milan	Mechanical ventilation with acute respiratory distress correlates with a significant higher BMI above 29.9. Acute respiratory distress and male sex are associated with obesity class I to III.	Chiumello et al. 2020
**176** **114** **59**	<30 >30	mortality *p* = 0.077 higher age of surviving patients *p* = 0.007	Greece/Athen	Type 2 diabetes and obesity are risk factors for disease severity and mortality in critically ill COVID-19 patients.	Halvatsiotis et al. 2020

BMI, the body mass index; ICU, intensive care unit, OR, odds ratio; MV, mean value.
